# Glycan masking in vaccine design: Targets, immunogens and applications

**DOI:** 10.3389/fimmu.2023.1126034

**Published:** 2023-03-23

**Authors:** Cristina E. Martina, James E. Crowe, Jens Meiler

**Affiliations:** ^1^ Department of Chemistry, Vanderbilt University, Nashville, TN, United States; ^2^ Center for Structural Biology, Vanderbilt University, Nashville, TN, United States; ^3^ Vanderbilt Vaccine Center, Vanderbilt University Medical Center, Nashville, TN, United States; ^4^ Department of Pediatrics, Vanderbilt University Medical Center, Nashville, TN, United States; ^5^ Department of Pathology, Microbiology and Immunology, Vanderbilt University Medical Center, Nashville, TN, United States; ^6^ Institute for Drug Discovery, Leipzig University Medical School, Leipzig, Germany

**Keywords:** glycan masking, vaccine design, reverse vaccinology, immuno-focusing, immuno-shifting

## Abstract

Glycan masking is a novel technique in reverse vaccinology in which sugar chains (glycans) are added on the surface of immunogen candidates to hide regions of low interest and thus focus the immune system on highly therapeutic epitopes. This shielding strategy is inspired by viruses such as influenza and HIV, which are able to escape the immune system by incorporating additional glycosylation and preventing the binding of therapeutic antibodies. Interestingly, the glycan masking technique is mainly used in vaccine design to fight the same viruses that naturally use glycans to evade the immune system. In this review we report the major successes obtained with the glycan masking technique in epitope-focused vaccine design. We focus on the choice of the target antigen, the strategy for immunogen design and the relevance of the carrier vector to induce a strong immune response. Moreover, we will elucidate the different applications that can be accomplished with glycan masking, such as shifting the immune response from hyper-variable epitopes to more conserved ones, focusing the response on known therapeutic epitopes, broadening the response to different viral strains/sub-types and altering the antigen immunogenicity to elicit higher or lower immune response, as desired.

## 1 Introduction

Carbohydrates are one of the basic building blocks of the cell, together with nucleotides, amino acids and lipids (1). Complex sugar chains (or glycans) can be covalently attached to proteins (forming glycoproteins), to lipids (glycolipids), and also to RNA (glyco-RNA) (2). The covalent link between a glycan chain and a protein can be mediated by nitrogen atoms (referred as N-glycosylation), by oxygen atoms (O-glycosylation), or, more rarely, by sulfur atoms (3).

In eukaryotes, glycan chains are added to a nascent polypeptide by the glycosyltransferases in the endoplasmic reticulum (ER) (4). These enzymes recognize a pattern of residues on the unfolded protein, called sequon, that is specific for each type of glycosyltransferase and glycosylation (N or O-linked) (5). The sequon for N-glycosylation, used in glycan masking, is formed by the triplet NxS/T, in which the first residue is the asparagine that will be glycosylated, the second can be any amino acid except for proline and the third is a serine or a threonine (6).

Following glycosylation in the ER, the protein is then transported through the Golgi apparatus, in which other glycosyltransferases and glycosidases can add, remove or modify the basal glycosylation (4). After modification, the protein is then exported to the surface of the cell or released in the extracellular matrix.

The mechanism of action of the glycosyltranferases in the ER or in the Golgi is not fully understood. The same asparagine in a protein can be found with different sugar types, a phenomenon referred as glycan heterogeneity (7). For example, the SARS-CoV-2 spike protein presents a total of 183 different glycoforms, 114 of which on the same glycosylation site (8).

Most enveloped viruses such as influenza, HIV and SARS-CoV-2 present one or more glycoproteins exposed on the surface of the viral nanoparticle (9–11). These proteins are involved in receptor binding and cell entry, and they are one of the main target of the immune response during viral infection (12). In order to escape recognition from the immune system and in particular from therapeutic antibodies, viruses undergo rapid mutations which alter their glycoproteins immunogenicity (13). The insertion of new glycosylation sites on the protein surface blocks recognition and antibody binding, leading to lack in protection and increased vulnerability during infection. This natural phenomenon of glycan shielding, or glycan masking, has been adapted to alternative applications, such as immuno-focused vaccine design or protein-protein interface mapping (14, 15).

In this review, we will give an overview of the state-of-the-art in glycan masking, highlighting successes and limitations of this technique. We will describe the pathogens and antigens considered for epitope focused immunogen design and the vector systems used for enhancement of the immune response. Finally we will then explain the different applications that can be accomplished through glycan masking, including immuno-shifting, immuno-focusing, immuno-broadening and immuno-altering.

## 2 Literature overview

We selected 25 scientific articles in which glycan masking has been applied to vaccine design. Selection of the papers is based on: 1- the use of N-glycosylation through the insertion of a NxT/S sequon; 2- the addition of non-native glycans to the protein of interest; 3- the creation of immunogen candidates. The 25 selected articles are listed in publication order in **Table 1**. Articles published by the same research group, and with same pairs of targets and immunogens are grouped within a single row in the table for a total of 19 independent references.

**Table 1:** Collection of articles on vaccine design through glycan masking.

Ref.	Pathogen, Antigen	Immunogen	Shifting	Focusing	Broadening	Altering	Other
Garrity et al. (16)	HIV, Env	Env, vaccinia virus	Dominant		HIV-1 IIIB, MN		
Pantophlet et al. (17) Pantophlet et al. (18) Selvarajah et al. (19) Ahmed et al. (20)	HIV, Env	Gp120 (monomer)		Receptor Binding Site	HIV clades A, B, C		
Selvarajah et al. (21)	HIV, Env	sEnv (trimer)	Dominant		HIV clades A, B, C		
Wanzeck et al. (22)	Influenza, HA H3	HA, influenza virus					Simulate infection
Lin et al. (23) Lin et al. (24) Chen et al. (25)	Influenza, HA H5	HA, FliC, VLP sHA, ADV sHA	Variable		Flu H5 sub-clades		
Sampath et al. (26)	Malaria, DBP	DBP domain II	Polymorphic			Increase	Understand binding
Forsell et al. (27)	HIV, Env	sEnv (trimer)	Dominant				B-cell regulation
Eggink et al. (28)	Influenza, HA H1	HA, sHA	Dominant		Flu H1, H5		
Ingale et al. (29)	HIV, Env	Engineered gp120		Receptor Binding Site	HIV groups 1 and 2		
Du et al. (30)	Coronavirus, Spike	RBD (monomer)					Neutralization score
Kulp et al. (31)	HIV, Env	Engineered Env	Artificial				Off-target response
Duan et al. (32)	HIV, Env	Engineered gp120, NP		Receptor Binding Site		Decrease	Antibody precursors
Bajic et al. (33) Thornlow et al. (34)	Influenza, HA H3	sHA, HA1	Dominant	Receptor Binding Site	Flu H3, H4		
Lin et al. (35)	ZIKV, DENV, E protein	E domain III, FliC		Therapeutic Epitope			ADE activity
Tai et al. (36)	ZIKV, E protein	E domain III	Artificial			Decrease	
Boyoglu-Barnum et al. (37)	Influenza, HA H1	Engineered HA2, NP		Conserved Domain	Flu H1, H3, H5, H7, H10		
Chen et al. (38)	Influenza, HA H1	HA, ADV	Variable		Flu H5 sub-clades		
Adolf-Bryfogle et al. (39)	Nanoparticle	NP	Artificial			Decrease	
Lin et al. (40)	Coronavirus, Spike	Spike, ADV			SARS-CoV2		

VLP, virus-like particle; ADV, adenoviruses; NP, nanoparticle.

We acknowledge that successful immunogen design examples were obtained also through removal of native glycans from the antigen of interest, referred as glycan unmasking (41, 42). Moreover, an interesting alternative in epitope masking is the nanopatterning technique, in which polyethylene glycol (PEG) molecules are used instead of glycans (43, 44). Finally, glycan masking was also successfully applied to the design of protein-based drugs such as interferon alpha 2 (45), human coagulation factor IX (46, 47) human erythropoietin (48) and human hormones (49, 50). Glycan unmasking, nanopatterning and glycosylated drugs however are out of the scope of this review and thus are not included in our collection.

## 3 Pathogens and antigens in glycan masking

Immunogen design through glycan masking is not trivial: prior knowledge on protein structures and antibody response is essential, and multiple rounds of experimental validation are needed to obtain valid vaccine candidates. For these reasons, glycan masking is applied only to historically challenging pathogens, for which conventional vaccination methods are not available or failed to completely eradicate them. The majority of the pathogens described in our collection are viruses: seven out of the 19 references target human immunodeficiency virus (HIV), six influenza virus, two coronavirus and two Zika and Dengue viruses. The only non-viral pathogen included in our collection is the parasite *P. vivax*, involved in malaria disease. These pathogens are extremely challenging due to a variety of reasons, including the ability to quickly evolve, the propensity to escape the immune system, the existence of a pathogenic reservoir in different host species and the absence or inefficacy of current vaccines.

The selected antigens for glycan masking are proteins exposed on the surface of the pathogens in 18 out of the 19 references. This is not surprising, since the surface proteins can directly be recognized and targeted by the antibodies produced by the immune system. The only reference not associated to human pathogens is (39), in which the glycosylated protein is a self-assembling nanoparticle developed to better expose immunogen candidates to the immune system (39).

### 3.1 HIV and Env protein

HIV has been discovered in 1983 and it caused more than 36 million deaths worldwide (51). HIV-1 group M is responsible for the majority of HIV infections and it includes nine genetically different sub-types (A, B, C, D, F, G, H, J and K) and multiple hybrids (circulating recombinant forms, CRFs) (52). Sub-types A, B and C account for more than 70% of the global prevalence for HIV world-wide and represent the major focus in the development of therapeutics.

Efforts in producing an effective vaccine against HIV have been taken for decades (53), however no vaccine has been approved by the Food and Drug Administration (FDA) yet. Major difficulties in vaccine development have been associated to: 1- the co-existence of multiple HIV groups and sub-types; 2- the viral ability to rapidly mutate and escape the immune system and 3- the persistency of viral reservoir in the host immune system (54).

The HIV surface glycoprotein is Env, a trimer of heterodimers composed by a N-terminal ectodomain (gp120) and a C-terminal membrane domain (gp41) (55). Env is initially produced as a single polypeptide of 160 kDa, referred as gp160, that is then cleaved during viral maturation. Gp120 includes five conserved regions (C1-C5), which mediates binding to the host receptor CD4, and five variable regions (V1-V5). Gp41 is composed by a cytoplasmic domain, a transmembrane domain and an extracellular domain which drives cell fusion and entry. The Env protein is heavily glycosylated, with 18 to 33 native glycans per monomer (56). Glycosylation and mutations accumulated in the variable regions of gp120 mainly contribute to viral escape (54).

### 3.2 Influenza virus and HA protein

Influenza virus caused countless epidemics and three major worldwide pandemics in the last century: the Spanish flu in 1918, with 50 million deaths; the Asian flu in 1957, with 1 million deaths and the Hong Kong flu in 1968, also with 1 million deaths (57). Influenza virus is classified in four types, A, B, C and D, with only the first three causing illness in humans. Influenza A and B are cause of multiple epidemics, but only type A is cause of the pandemics. Influenza A is divided in sub-types based on the two surface glycoproteins: hemagglutinin (HA) and neuraminidase (NA). There are 18 different HA sub-types (H1-H18) and 11 NA sub-types (N1-N11), but more than 130 combinations of HA and NA have been identified (57). Influenza virus can undergo to two main evolutionary processes: antigenic drift, in which mutations are accumulated overtime leading to viral escape, and antigenic shift, in which co-infection of avian, swine or human influenza variants led to gene reassortment (59). Antigenic drift and shift highly contribute to the long-term inefficiency of current vaccinations against influenza virus. Efforts in vaccine design have mainly focused on HA, since it is responsible for cell attachment and entry (60).

HA, similarly to the Env protein of HIV, is a trimer of heterodimers formed by a head domain (HA1) and a stem domain (HA2), also referred as stalk. The head domain engages and binds the host receptor (sialic acid) through the conserved Receptor Binding Site (RBS), while the stem domain is involved in membrane fusion and cell entry mediated by the fusion loop (61).

The majority of the antibodies elicited by influenza infection or vaccination target the HA head domain (62). For this reason, the head domain is considered immuno-dominant, while the stalk domain is considered immuno-recessive. However, the head domain is also highly variable and mutagenic, and antibodies directed toward the head domain are in general less broadly reactive to other viral strains than the antibodies directed towards the more conserved stalk.

N-glycosylation of HA plays a key-role in receptor binding, membrane fusion, virulence and escape (63). Glycans located in the stem domain are necessary for proper folding and trimerization, and are highly conserved among different viral strains. On the other hand, glycans located on the head domain are mainly involved in viral escape and they widely differ in number and location: for example the HA head domain of influenza H3N2 counts from two to six glycosylation sites, while H7N9 has a single glycosylation site (64).

### 3.3 Coronavirus and Spike protein

The coronavirus family includes three main viruses that caused disease in humans: SARS-CoV, which originated the SARS outbreak in 2002-2003 with 8000 infections and at least 774 deaths; MERS-CoV, which caused outbreaks in 2012, 2015 and 2018 with more than 2500 cases and 880 deaths (65) and SARS-CoV-2, which caused the global pandemic in 2019, currently ongoing, with more than 600 million cases and 6,4 million deaths worldwide (66).

In only two years SARS-CoV2 accumulated hundreds of different mutations in its genome, and five variants are considered a major concern: Alpha, first described in UK in December 2020; Beta, South Africa in December 2020; Gamma, Brazil in January 2021; Delta, India in December 2020 and Omicron, South Africa in November 2021 (67).

In 2020-2022, 18 different vaccines have been developed against the original strain of SARS-CoV2 (Wuhan-Hu-1): three are RNA-based, four protein-based, four non-replicating viral vectors, and seven are inactivated virus (68). Most vaccines result effective against newer viral variants including Alpha, Beta, Gamma, Delta and Omicron (69), but it is not clear yet if they will be protective against new emerging strains of SARS-CoV2.

The spike protein, also referred as protein S, is the major target in antibody response upon both natural infection and vaccination protocols (70). As for HIV and influenza virus, the spike protein is a trimer of heterodimers composed by the S1 and S2 domains. The receptor binding domain (RBD), mediates cell attachment by binding to the ACE2 receptor (SARS-CoV and SARS-CoV2) or to the DPP4 receptor (MERS-CoV). The RBD is located in S1, together with the N-terminal domain (NTD) and the C-Terminal domain (CTD). The S2 subunit includes the Fusion Peptide (FP), two heptad repeat regions, the single-pass transmembrane domain and the cytoplasmic tail. FP mediates fusion of the host and virus membranes, allowing viral entry in the cells (11).

The SARS-CoV2 Spike protein contains native 22 glycosylation sites per monomer, mostly located at the NTD domain in the S1 sub-unit (71).

### 3.4 Dengue virus, Zika virus and E protein

Dengue and Zika viruses belong to the same Flaviviridae family and, together with malaria, they represent the major health concern for mosquito-borne diseases.

Dengue infection rates increased dramatically in the past decades, with 50-100 million cases every year. Mortality is lower than HIV or influenza virus, with a global average of more than 9000 cases per year (72). Dengue virus is divided in four main serotypes (DENV1-4) and 19 genotypes with different transmission rates, cellular targets and immune responses. Antibodies produced after primary infection are able to cross-react, but not neutralize, other Dengue strains, and during secondary infection they might cause more severe symptoms and illness. This phenomenon, called Antibody Dependent Enhancement (ADE), represents one of the main limitation in the production of effective vaccines against dengue virus (73). There is a single licensed vaccine against dengue virus, named Denvaxia, but it is currently administered only to individuals that have already been infected naturally by the virus, while seronegative individuals have higher risk of developing severe illness after secondary infection (74).

Zika virus outbreaks were recorded in 2007 in the Pacific Islands and in 2015-2016 in Latin America, with an estimation of 1.62 million cases (75). Zika infections in adults lead generally to mild symptoms, however the virus can be transmitted from pregnant women to fetuses causing miscarriages, congenital microcephaly, brain malformations or eye abnormalities in the newborns (76). No vaccine is currently available against Zika virus.

The envelope protein of dengue and Zika virus is the E protein, and it is involved in receptor binding and cell fusion. Differently from the Env, HA and Spike proteins which belong to class-I fusion proteins, E protein belongs to the class-II, and in pre-fusion state it forms a head-to-tail homodimer. Each monomer is formed by five domains: a finger-like dimerization domain containing the fusion loop (DII), a beta-barrel domain (DI), an immunoglobuline like domain (DIII), a stem domain and a transmembrane domain (77). DIII, from both dengue and Zika, has been reported to elicit neutralizing antibodies (nAbs) in animals and represent a major target for immunogen design (78, 79). Dengue E protein contains two glycosylation sites per monomer, while Zika E protein only one. Glycosylation plays a major role in cell attachment viral replication, transmission in both mammalian and insect cells and overall pathogenesis (80).

### 3.5 Plasmodium vivax (Malaria disease)

The only parasite included in our collection is *Plasmodium vivax*, one of the six *Plasmodium* variants responsible for malaria disease (81). Malaria circulated globally since millennia, with 300 million deaths in the 20th century and over 600000 in 2020 (82). Challenges in the eradication of malaria disease include the parasite complex multi-stage life cycle, its persistency in mosquito reservoir and the development of resistance against drugs and insecticides (83).

Antibody-mediated immune response against *P. vivax* was shown in 1961 when purified immunoglobulines were passively transferred from adult survivors to young children, showing protection (84). This discovery raised interest in the development of vaccines, however only one candidate, PfSPZ, reached clinical trial phase IV and is currently in trial until 2023 (85).

The Duffy Binding Protein (DBP) is the surface protein selected for malaria-based immunogen design described in Sampath et al. (26). DBP mediates attachment, entry and invasion of red blood cells, essential steps in the parasite life cycle. DBP binds to the host DARC receptor with region II, formed by 3 subdomains (86). Epitopes reported to be highly immunogenic are the dimeric interface, the conserved subdomain 3 and the subdomain 2 (87).

## 4 Immunogens in glycan masking

Epitope-focused immunogen design through glycan masking is based on the idea that specific epitopes, known for eliciting therapeutic antibodies, are fully accessible for recognition by the immune system, while other non-relevant epitopes are hidden with glycan chains. To mask a specific epitope, selected residues have to be substituted with the sequon for N-glycosylation, NxS/T where x can be anything except for a proline (6). The sequon is necessary for insertion of glycan chains, however refined databases for glycoproteins showed that not all sequons are glycosylated once the protein is produced within the cell (88). This possibility has to be kept in mind while designing immunogen candidates.

Moreover, careful preparation of the immunogens is essential to maximize the exposition of therapeutic epitopes and to minimize the availability of non-therapeutic ones. Three main categories of immunogens are used in our collection: native-like, corresponding to the full extracellular antigen exposed by the pathogens; domain-based, in which only a portion of the antigen is selected for glycan masking and engineered antigens. All types of immunogens can be “loaded” onto a supra-molecular vector systems, which better expose the epitopes to the immune system. All four cases will be described in this chapter.

### 4.1 Native-like immunogens

Native-like immunogens created through glycan masking include both the full antigen proteins or their soluble versions, which lack the intracellular and transmembrane domains. The advantage to use native-like immunogens is that the overall structure closely mimic the one exposed on the pathogens surface, including their oligomeric state. Moreover, the risk to present artificial epitopes is minimal, thus avoiding the production of off-target antibodies. Disadvantages however are multiple: some antigens are poorly stable when lacking the transmembrane and intracellular domains, or they fail in forming the correct oligomeric complex with high efficiency. Moreover many viral glycoproteins are heavily glycosylated in their native form (i.e., HIV). The addition of numerous non-native glycans during glycan masking might lead to misfolding and non-expression of the immunogen candidate (89), or to significant decrease in immune response upon immunization.

Immunogens created against HIV presents the trimeric env protein in its native conformation, using the full gp160 gene (16), or gp140 (referred also as sEnv), a soluble version of gp160 lacking the intracellular and transmembrane domains (21, 27). Immunogens against influenza virus present the full trimeric HA antigen, including the intracellular C-terminal region and the transmembrane domain (22), or the soluble ectodomain version sHA (33, 38), or both (23–25, 28). Immunogens against SARS-CoV2 are also composed by the full length Spike protein in its native conformation (40).

### 4.2 Domain-based immunogens

Differently from the native-like immunogens, the domain-based ones are composed by a sub-portion of the full antigen: the gp120 domain of Env of HIV (17–20), the subdomain II of DBP of *P. vivax* (malaria) (26), the RBD of the spike protein of MERS (30), the HA head domain (HA1) of hemagglutinin of influenza virus (33, 34) and the EDIII domain of (35, 36) viruses.

Domain-based immunogens are generally easier to produce due to their smaller size and do not require *in vitro* oligomerization; however they might present artificial epitopes which can elicit off-target antibodies. Generally, the choice of a domain-based immunogen is driven by prior knowledge on the overall antibody response against the full antigen: if a domain is associated with higher protection, removing the other poorly therapeutic domains contributes to better focus the immune system and to obtain more protective antibodies.

In some cases, the choice of a domain-based immunogen is driven by the lack of structural knowledge for the full antigen, and only individual domains are available to guide the epitope-focused immunogen design through glycan masking.

### 4.3 Engineered immunogens

Engineered versions of the antigens are used as immunogens in four cases out of our 19 references. These variants are mostly used for increased stability and expression yields, for decreased immunogenicity, or to mimic specific conformations. Extensive research is necessary to generate engineered proteins prior to glycan masking.

The glycan masked candidates designed in (29) against HIV-1 are based on Ds12F123 core, and engineered version of gp120 in which the major variable loops V1, V2 and V3 have been deleted and mutations have been introduced to adopt a CD4-bound conformation (90).

Similarly, the immunogen candidates against HIV created by (31) are based on BG505 SOSIP.664, an engineered version of gp140 with superior expression yields and stability, and with mutations in the variable loop V3 to reduce reactivity (91). Immunogens from Duan et al. against HIV (32) are based on eOD-GT8 60-mer, a circularly permutated version of gp120 in fusion with a self assembling nanoparticle subunit (92).

Finally, Boyoglu-Barnum et al. developed an engineered version of HA in which only the stem portion is displayed in a trimeric conformation on a nanoparticle vector (37, 93). The HA stem is more conserved across different influenza strains than the HA head domain, and thus could elicit more broadly reactive antibodies.

### 4.4 Vector systems for immunogen display

One key factor to consider in vaccine design through glycan masking is how immunogens will be presented to the immune system. Although domain-based immunogen candidates are able to elicit an immune response, the use of adjuvants or delivery systems was shown to enhance the immune response (94).

Full native-like immunogens containing the transmembrane domain require a membrane environment for stability. Using the enveloped virus itself is a valid option when the risks of infection lead to less severe outcomes. For example, the glycosylated version of the full HA of influenza virus developed by Wanzeck et al. has been reintroduced in the influenza genetic material, and the full virus has been used for animal immunization (22).

However, this would not be possible with pathogens that cause highly severe symptoms or terminal illness, such as HIV. To overcome this limitation, Garrity et al. used recombinant vaccinia virus, an enveloped virus widely used in human vaccination (95), to expose the full glycosylated Env protein and immunize animal models (16).

Adenoviruses (ADVs) are another class of non-enveloped viruses widely used for vaccination (96). They are used to expose the full glycosylated version of the Spike protein of SARS-CoV2 (40), however, due to the fact they do not have a membrane envelope, they are more often used with soluble versions of the native antigen (lacking the transmembrane domain) or with domain-based immunogens. The soluble glycosylated versions of HA have also been exposed on an adenovirus particle (24, 38).

Virus-like particles (VLPs) are great alternatives to viral vectors for vaccination purposes (97). VLPs are nanoparticles formed from viral proteins, but lacking the genetic material, and thus not able to replicate. They better mimic the dimension of a viral pathogen and are safer than live virus systems. VLPs are used to expose the full glycosylated HA and immunize mice (23).

Similarly to VLPs, self-assembling non-viral nanoparticles (NPs), are used as vector system to better expose immunogen candidates. This is the case for the engineered Env immunogen against HIV (32) and for the engineered HA stem against influenza virus (37).

A major difficulty in using non-viral systems or proteins, is that the immune response upon immunization can be directed not only against the immunogen itself but also against the vector system. In particular, a fraction of the antibodies produced by the immune system would be directed toward an artificial site, which would not be present on the pathogen of interest. To overcome this problem, Adolf-Bryfogle et al. performed glycan masking on a two-component self-assembling nanoparticle specifically designed to expose immunogen candidates (98, 99). Although immunogen candidates are not present on the nanoparticle itself, the glycosylated vector system elicited a lower immune response after immunization in comparison to the unglycosylated version, showing that glycans were able to hide the artificial epitopes (39).

Finally, the only other vector system used in our collection is the soluble form of the flagellin protein (FliC), which has been considered as vaccine adjuvant to enhance the immune response (100). FliC has been used in fusion with the full HA and exposed on a VLP (39), and in fusion to the DIII domain of the E protein of Dengue and Zika viruses (35).

## 5 Glycan masking, main goals and strategies

As implied in the name, one of the main application of glycan masking is to “mask” or “hide” unwanted portions of the protein of interest. However different goals can be reached with the same technique. Among the 19 references, we identified four main categories for which glycan masking is widely used: immuno-shifting, immuno-focusing, immuno-broadening and immuno-altering (**Table 1**). Goals that are specific for a single reference are grouped in the fifth category “other goals” and are described in the next chapter.

### 5.1 Immuno-shifting

The most common reason to use glycan masking in immunogen design is to shift the immune response from certain epitopes to others. Immuno-shifting can be accomplished also with other techniques, from simple mutagenesis to deletion of larger regions of the protein. For example, glycosylations are added to an engineered version of the env proteins in which the variable regions were previously removed or mutated (29, 31, 90, 91). Immuno-shifting mediated by glycosylation is performed in 11 out of the 19 references presented in this review. Most commonly the goal of glycan masking is to shift away from immuno-dominant epitopes, which elicit high antibody titers but low neutralization activity and thus low protection. Such epitopes are also called “immune decoy epitopes” or “decotopes” by (16).

Immuno-shifting is also used for antigens with highly variable regions, which might elicit potent immune response, but not a broader one. To note, most of the highly variable epitopes considered in our references are also immuno-dominant, however not all the immuno-dominant epitopes are highly variable: the Receptor Binding Site (RBS) of HA is an example of conserved epitope in an immuno-dominant region. Immuno-recessive regions are usually associated to more conserved epitopes, able to elicit broadly neutralizing antibodies.

For the env protein of HIV, immuno-dominant and highly variable epitopes are the V1, V2 and V3 loops. V3 only can be mutated to insert additional glycosylations (16, 27), or all three loops can be glycosylated (21). For influenza virus, the head domain of HA is the immuno-dominant domain and glycosylation was used to shift immune-response to the more conserved stalk (28, 33). The hyper-variable epitopes within the head domain were also mutated for immuno-shifting (23, 38). For malaria, the only non-viral entry in our set, glycan masking was used to shift immune-response away from the polymorphic region (26).

Finally, immuno-shifting is also used to mask artificial sites, generally defined as epitopes which, during natural infection, are not encountered by the immune system. Such epitopes derives in most cases from modification of the natural protein, by truncation or by extraction of individual domains. Glycans were added at the base of the env protein, a region that in the full virus is inaccessible due to the presence of the membrane and of the transmembrane region (31). In the work of Tai et al., the DIII domain of the protein E is used as recombinant immunogen candidate, and glycosylation is used to mask the protein side previously in contact with the other domains in the glycoprotein. Such site is not accessible during natural infection with Zika virus (36). Artificial sites include also proteins that were artificially designed, as for the nanoparticle used in (39). The nanoparticle is intended as a vector to present immunogens to the immune system, not as an immunogen itself, thus representing an artificial site.

### 5.2 Immuno-focusing

Alternative to immuno-shifting is immuno-focusing in which glycans are strategically added to focus the immune system to a particular epitope. More generally, immuno-shifting is best described as eliciting immune response “away from” certain epitopes, while immuno-focusing as eliciting it “to” specific epitopes.

In immuno-focusing, glycosylation sites are inserted outside the epitope of interest, ideally to reduce the production of off-target antibodies. Out of the 19 references we listed, six performs immuno-focusing. The major targets in this category are highly conserved epitopes able to elicit broadly neutralizing antibodies, as for the receptor binding sites CD4bs in HIV Pantophlet et al. (17–20, 29, 32, 33, 37). Glycan masking was used to focus on the DIII domain of Zika virus, previously shown to elicit broad neutralizing antibodies (35).

### 5.3 Broadening immune response

Immuno-broadening is defined as the ability for a given immunogen to give immune protection among a wider range of viral strains or pathogenic sub-types. Such wider protection is generally obtained when broadly neutralizing antibodies are elicited by highly conserved epitopes. Thus, both immuno-shifting and immuno-focusing can lead to immuno-broadening by respectively shifting or focusing the immune response to conserved epitopes. It is important to note that immuno-shifting or immuno-focusing can also lead to other outcomes rather than immuno-broadening, as seen in the next sections.

From a biomedical point of view, the ability to target multiple viral strains with a single vaccine is of high interest, and in nine out of the 19 references the goal of glycan masking is to broaden the immune response. In five cases immuno-broadening is reached by immuno-shifting: shifting away from the hyper-variable regions of HIV-1 (16, 21) and influenza virus (23–25), or by shifting away from the immuno-dominant epitopes in the HA protein of influenza (28). In three cases immuno-broadening is reached by immuno-focusing: for HIV-1 by focusing on the conserved receptor binding site, CD4bs (17–20, 29), and for influenza virus by focusing on the conserved stalk domain (37). Immuno-broadening for influenza virus is also attempted by both immuno-focusing on the receptor binding site (RBS) with one glycan mutant and by immuno-shifting away from the RBS with another mutant (33, 34).

### 5.4 Altering immunogenicity

Immuno-shifting, immuno-focusing and immuno-broadening are intended to change the immune response by altering the antibody recognition pattern, but not the overall amount of elicited antibodies. In other words, antibody titers obtained by immunization with the glycan mutants should remain the same as the ones obtained with the non-glycosylated protein. In immuno-altering the goal is to change the amount of total elicited antibodies. In case of poorly immunogenic proteins, such as the DBP protein of malaria (26), the goal of glycan masking is to increase antibody titers to obtain a more immunogenic protein. In case of immunogens that elicit good antibody titers, but that lack in therapeutic effects, such as the gp120 protein of HIV-1 Kulp et al. (31), the goal is to decrease the overall antibody titers and in particular to decrease the off-target ones. In case of immunogens presenting artificial sites that wouldn’t be encountered by the immune system during natural infection, such as the “hidden” epitope in the Zika virus immunogen (36), the goal is again to decrease the number of off-target antibodies. Finally, for completely artificial constructs such as the nanoparticle designed to better expose viral immunogens (39), the goal is to decrease the overall antibody titers, making it poorly immunogenic and focus the immune response to the loaded immunogens.

## 6 Glycan masking, other applications

Immuno-shifting, focusing, broadening and altering are goals shared by multiple references in our set, and they represent the major applications of immunogen design through glycan masking. However the technique can be applied also to answer very specific questions, as seen in seven references out of the 19.

### 6.1 Simulate natural infection to recent and ancient viral strains

Wanzeck et al. used glycosylation to study what happens if subjects immunized with hyper-glycosylated viral variant encounter poorly glycosylated ones (22). For example, ancient strains of influenza virus were initially poorly glycosylated and HA accumulated glycosylation during co-evolution with the human host to escape the immune system (64). The risk that ancient (and poorly glycosylated) influenza variants re-emerge among the human population is a major concern. In particular, a novel H1N1 influenza strain caused an outbreak in 2009 in Mexico, Canada and USA, leading to severe symptoms in younger patients and mild ones in elders (101). Wanzeck et al. replicated the H1N1 outbreak in mice, with the hypothesis that older patients encountered poorly glycosylated variants of influenza virus during their youth, and thus were protected, while younger patients were more susceptible because they only encountered hyper-glycosylated viral versions. First they immunized mice with an hyperglycosylated HA variant of influenza virus, and then they infected them with a hypoglycosylated virus. The authors were able to demonstrate that mice were more susceptible to the secondary infection against poorly glycosylated variants despite vaccination (22).

### 6.2 Understand mechanism of receptor binding

Sampath et al. used glycan masking to both shift and alter the immune response against the DBP protein of malaria, but also to infer the mechanism of binding of DBP to the human DARC receptor (26). The receptor is extremely important for the progress of the infection and DARC-null individuals are less likely to get sick (102). The underlying question is why DBP is poorly immunogenic. Two theories, non mutually exclusive, suggest that 1- antibody recognition is lacking due to a strong affinity between monomeric DBP and the DARC receptor and that 2- interactions with the DARC receptor induce dimerization of DBP thus limiting antibody binding to the conserved epitope (103). With the glycosylated immunogens the authors were able to block DBP binding to DARC receptor and individuate the binding interface (26).

### 6.3 Understand mechanism of B-cell regulation and competition

Forsell et al. used glycan masking to study how monoclonal B cell lineages develop, compete and persist *in vivo* after immunization (27). In particular they want to understand if B cell clones targeting independent epitopes compete with each other and if masking one epitope leads to overall reduction in the total immune response or if it leads to its redistribution to other epitopes on the antigen. Such knowledge is important for the design of epitope-focused vaccines (104). They use as model the soluble trimeric env protein of HIV (105) and insert three glycosylation sites to mask the V3 loop. Before mice immunization, they check binding of the wild-type and glycosylated antigens to plated naive B-cell, showing that the glycosylated immunogens have lower binding compared to the wild-type. This suggest that the available B-cell population for development of potent antibodies is reduced beforehand by the presence of the glycans. After mice immunization they confirm that different epitopes are independently regulated and that there is no competition among their B-cell lineages (27).

### 6.4 Score neutralization capabilities for different epitopes

Glycan masking has been used to develop the Neutralizing Immunogenicity Index (NII), a novel concept to quantitatively determine how different epitopes contributes to viral neutralization (30). This concept is very important in the field of immunogen design, since it can differentiate epitopes with a strong therapeutic response from the ones without (106). To calculate the NII value for each epitope, the neutralization activity of post-immunization sera is measured for both a wild-type and single glycan immunogens. Negative values of NII indicates that the epitope contributes negatively to the overall neutralization capability of the immunogen, while positive values indicates that the epitope contribute substantially to the therapeutic effect. Knowing the NII for each epitope in an antigen can easily guide the design of strong immunogens, since the ones contributing negatively in the NII scale can be masked or altered.

### 6.5 Test off-target immune response

Glycan-masked variants have also been used to probe the immune response elicited with standard (non glycosylated) immunogens, and in particular to identify the species-specific off-target response (31). A gp41-dependent, non neutralizing antibody was elicited in rabbits during immunization (107), and the glycan masked variants were able to partially or completely block its binding. A specific class of neutralizing antibodies (VRC01-like) targeting the CD4bs epitope were elicited in mice after immunization, together with off-target antibodies (108). The glycan masked variants were shown to block this off-target response without affecting the therapeutic response obtained by VRC01-like antibodies. Finally, sera obtained after immunization of non-human primates was tested in a competition assay with the glycan variants previously shown to block off-target response (109). Only 77% to 63% of reactivity was retained by the masked variants, suggesting that an off-target response might be elicited also in non-human primates.

### 6.6 Elicit antibody precursors

Duan et al. designed glycosylated variants to test for binding with both revertants and precursor of the broadly-neutralizing antibody VRC01 (32). The rational is that to elicit VRC01-like antibodies, the immunogen candidate must be able to also bind the VRC01 precursors. Immunogens based on the monomeric version of gp120 and on the native env trimer fail to bind VRC01 precursors, and consequently are not able to elicit this class of potent antibodies (110). Engineered antigen variants such as eOD-GT8 are able to bind both VRC01 precursors and revertants, however they display high off-target immunogenicity (91). To reduce the number of off-target antibodies glycans were strategically added on the surface of the engineered env trimer and tested for binding to VRC01 precursor and revertants. The glycosylated proteins were shown to produce up to 4 times more VRC01-like antibodies compared to the unmasked variant (32). The concept of testing for precursor binding is innovative in the glycan masking field and its integration in the immunogen design protocol could lead to better vaccine candidates.

### 6.7 Reduce antibody-dependent enhancement activity

Glycosylated immunogen candidates designed to mask the highly conserved fusion loop (FL) epitope in the DII domain of protein E of DENV and ZIKV were developed by (35). The FL epitope was shown to elicit cross-reactive non-neutralizing antibodies able to facilitate viral entry in the cell in secondary infections, thus enhancing the disease instead of preventing it Halstead (111). This phenomenon is called antibody-dependent enhancement (ADE), since the worsening of the disease is mediated by antibodies. The ADE activity has been shown for multiple pathogens, including DENV and ZIKV (73), and it is a major problem in the development of vaccine candidates (112). To mask the FL epitope, four immunogens were designed, each with one glycosylation. One out of the four immunogens was able to reduce ADE activity compared to the wild-type variant, while the remaining three were able to completely abolish it. This experiment demonstrates that glycan masking can be successfully used to prevent disease enhancing ADE (35).

## 7 Discussion

Glycan masking in epitope-focused immunogen design is a powerful technique, as shown in the 19 successful examples reported in this review. Upon immunization in animal models, the glycosylated immunogens were able to shift the immune response from immuno-dominant and hyper-variable epitopes to more conserved ones, to focus it on the epitopes of interest, to broaden it to alternative viral strains and to alter it by increasing or decreasing the overall antibody response. Glycan masking has also been used to reduce ADE activity, to elicit antibody precursors, to test off-target response, to evaluate the neutralizing capabilities for different epitopes, to inspect the B-cell regulation mechanism and to simulate potential viral outbreaks.

Immunogens in glycan masking span from native-like antigens, sub-domains or engineered candidates. Vector systems can be used to better display the immunogens, to improve their stability, or to enhance the immune response. Any pathogen could potentially be selected for glycan masking, however the current cases are limited to pathogens for which conventional vaccination failed or is not approved yet.

There are various reasons for which glycan masking cannot represent the first attempt of vaccine design given a new pathogen outbreak. First, knowledge on the immunogenicity of the pathogen extra-cellular proteins is necessary. Proteins associated with a strong immune response, i.e., potent neutralizing antibodies, are the ideal candidates for glycan masking. Second, glycan masking often requires multiple rounds of glycosylation to obtain successful candidates. Extra glycosylations can lead to misfolding, and protein expression is inversely correlated to the number of glycosylation added to the protein of interest. Moreover, the sequon sequence can be present at the correct location on the antigen, but the glycosylation could be missing. In this situation, the only solution is to re-try masking by picking nearby residues.

A third limitation in immunogen design through glycan masking is the availability of structural information. Knowledge on the overall atomic structure of the antigen is recommended to successfully identify potential glycosylation sites on the protein surface, and thus avoid misfolding. The lack of structural information, i.e., when only a domain is experimentally determined in a protein, might lead to introduction of artificial sites that can trigger an off-target immune response. This limitation can however be overcome with the advances in protein structure prediction lead mainly by AlphaFold (113) and RoseTTA Fold (114), in which accurate protein models can be obtained through machine learning methods.

## Author contributions

CM conceived and wrote the initial draft of the review. CM, JC and JM contributed to manuscript revision and all authors approved the submitted version.
